# Functional physiological phenotyping with functional mapping: A general framework to bridge the phenotype-genotype gap in plant physiology

**DOI:** 10.1016/j.isci.2021.102846

**Published:** 2021-07-10

**Authors:** Arun K. Pandey, Libo Jiang, Menachem Moshelion, Sanbon Chaka Gosa, Ting Sun, Qin Lin, Rongling Wu, Pei Xu

**Affiliations:** 1 College of Life Sciences, China Jiliang University, Hangzhou 310018, China; 2 Center for Computational Biology, College of Biological Sciences and Technology, Beijing Forestry University, Beijing 100080, China; 3 The Robert H. Smith Institute of Plant Sciences and Genetics in Agriculture, The Robert H. Smith Faculty of Agriculture, Food and Environment, The Hebrew University of Jerusalem, Rehovot 76100, Israel; 4 Biozeron Biotechnology Co., Ltd, Shanghai 201800, China; 5 Center for Statistical Genetics, Departments of Public Health Sciences and Statistics, The Pennsylvania State University, Hershey, PA 17033, USA

**Keywords:** Plant genetics, Plant biology, Omics

## Abstract

The recent years have witnessed the emergence of high-throughput phenotyping techniques. In particular, these techniques can characterize a comprehensive landscape of physiological traits of plants responding to dynamic changes in the environment. These innovations, along with the next-generation genomic technologies, have brought plant science into the big-data era. However, a general framework that links multifaceted physiological traits to DNA variants is still lacking. Here, we developed a general framework that integrates functional physiological phenotyping (FPP) with functional mapping (FM). This integration, implemented with high-dimensional statistical reasoning, can aid in our understanding of how genotype is translated toward phenotype. As a demonstration of method, we implemented the transpiration and soil-plant-atmosphere measurements of a tomato introgression line population into the FPP-FM framework, facilitating the identification of quantitative trait loci (QTLs) that mediate the spatiotemporal change of transpiration rate and the test of how these QTLs control, through their interaction networks, phenotypic plasticity under drought stress.

## Highlights

• FPP-FM allows real-time depicting of genetic interactions in response to environment
• It estimates genetic effects from physiological and developmental principles of growth
• FPP-FM serves the translation of physiological data into genetic gain

## Graphical abstract

**Figure:**
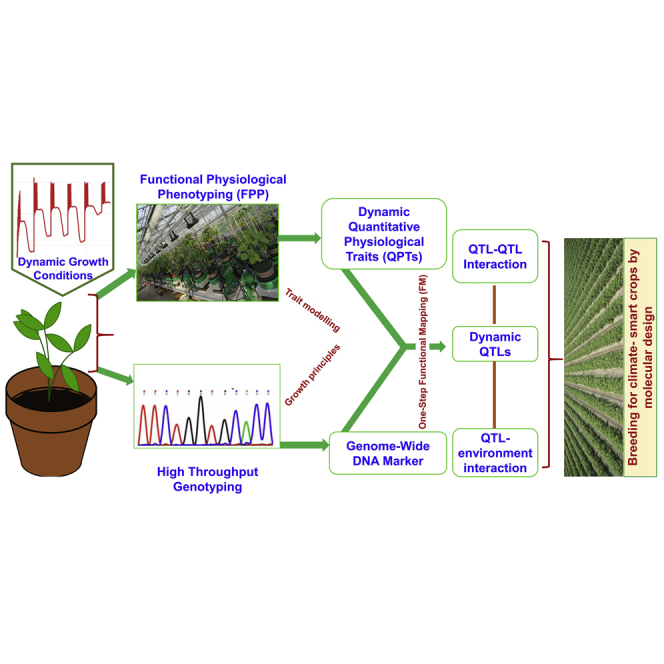

## Introduction

Plant phenotyping technologies have been described as the quantification of specific plant traits for research or breeding purposes that include growth, development, environmental responses, and other morphological and physiological changes (Araus et al., 2018). High-throughput phenotyping techniques built on automatic or semiautomatic platforms have emerged in recent years, providing the capacity to characterize structural and functional traits of plants from the cellular level to the whole plant or canopy level under controlled conditions (Tardieu et al., 2017). On the other hand, next-generation genotyping technologies have also progressed rapidly and provided great value in the field of genomic profiling and DNA sequencing (Fahlgren et al., 2015). Such advancement in technology is seeking to build an era of digital agriculture that is expected to significantly benefit plant researchers, breeders, and ultimately, consumers.

Drought is a severe agricultural problem facing plant scientists and farmers worldwide. From an agronomic perspective, drought resistance is defined by the capacity of a plant to sustain yield under water deficit during the life cycle (Negin and Moshelion, 2017). Physiological traits related to plant water relations are among the most relevant traits to agronomic drought resistance, whose fine-tuning would lead to optimized water management in response to drought stress (Blum, 2009; Sinclair et al., 2017). These traits include, for example, water use efficiency (Blum, 2009), transpiration-limited traits (Sinclair et al., 2017), midday water potential (Williams and Araujo, 2002), and stomatal conductance (Roche, 2015). Recently, the term “functional physiological traits” or “quantitative physiological traits” (QPTs) was coined to refer to these traits (Negin and Moshelion, 2017). Compared with morphological traits, which are defined as the changes to the outward appearance of a plant or the form and structure of internal parts (e.g. stem greenness, leaf wiltness), physiological traits are more sensitive to drought stress and thus provide a means of manipulating plant drought responses in an earlier stage before any morphological response is visible (Negin and Moshelion, 2017). In field conditions, plant drought responses are highly dynamic because the ambient environment is changing. Depending on the drought scenarios (mild or severe, transient, or long-term), a certain QPT may impact the yield positively or negatively. For example, low stomatal conductance is beneficial under severe drought conditions as it prevents excess water loss, but it may be undesired under mild drought as it constrains photosynthesis as well (Galkin et al., 2018). The property of an organism to produce distinct phenotypes in response to the environment is known as phenotypic plasticity (Davière and Achard, 2016; Ibañez et al., 2017). The degree of the phenotypic plasticity of quantitative physiological traits in plants can be determinant of their adaptive advantage to the environment (Nicotra et al., 2010; Pham and McConnaughay, 2014), thus regarded as being a valuable breeding trait related to food security (Gratani, 2014; Puglielli et al., 2017). However, it has never been easy to harness QPT plasticity. First, there are difficulties in precise phenotyping of yield-related QPTs due to their dynamic nature. A robust method requires a phenotyping system capable of continuously and simultaneously monitoring plant physiological traits responding to dynamic environmental fluctuations, in particular, soil-plant-atmosphere continuum (SPAC) parameters (Xu et al., 2015). Second, there is currently a huge phenotype-genotype knowledge gap to handle the massive quantitative physiological traits data acquired from pedigreed or natural populations to enable the identification of specific quantitative trait loci (QTLs) and their genetic interactions underlying phenotypic variations. Traditional genetic mapping methods deal with the time-series data each at a single time point (thus is “static”), usually failing to uncover the inherent dynamics of QTL effects (Li et al., 2015; Feldman et al., 2018; Guo et al., 2018). In addition, as the data volume increases radically in the phenomics era, traditional mapping may become technically intractable. There is hence an urgent need for more robust ways to integrate physiological knowledge, statistical genetics, and computational biology to dissect when (temporal) and where (spatial) a given genotype or allele has comparative advantages under stressful conditions.

Recently, several high-throughput phenotyping platforms tailored for physiological measurements have emerged to provide solutions to the aforementioned challenge in precise QPT phenotyping. Among them, platforms based on weighing lysimeter and gravimetric arrays are nondestructive, noninvasive systems enabling simultaneous and continuous monitoring of plant water relations under dynamic radiation, temperature, relative humidity, and vapor pressure deficit conditions (Vadez et al., 2015; Rymaszewski et al., 2017) Nevertheless, efficient genetic mapping of these longitudinal quantitative traits remains a major challenge, despite a few recent advancements in functional mapping (FM) using different modeling strategies such as Bayesian group lasso approach and estimating equations approach (Li et al., 2015; Li and Sillanpää, 2015). FM is a general statistical mapping framework proposed to characterize QTLs that underlie a complex dynamic trait (Li et al., 2015; Wu and Lin, 2006). It was developed based on the assumption of function-valued traits that phenotypic values expressed in the course of plant growth or treatment follow a continuous function/curve over time (Gomulkiewicz et al., 2018). By using mathematical equations to integrate the dynamic pathways behind the phenotypic formation, FM can unveil QTLs involved in rate-limiting processes and quantify the dynamic change of their genetic effects across a time or space scale (He et al., 2010; Li and Wu, 2010). From a statistical perspective, functional mapping estimates parameters that determine a genotype-specific growth or response curve and/or the covariance structure, thus strikingly increasing its statistical power for QTL detection (Sillanpää et al., 2012; Jiang et al., 2016). FM has shown remarkable power in associating QTLs with dynamic traits in plants and animals, such as growth trajectory, allometric scaling (Sillanpää et al., 2012; Jiang et al., 2016), biological shape (Sun et al., 2018), and drug or nutrient responses (Wang et al., 2013; Jiang et al., 2019).

**Figure 1:**
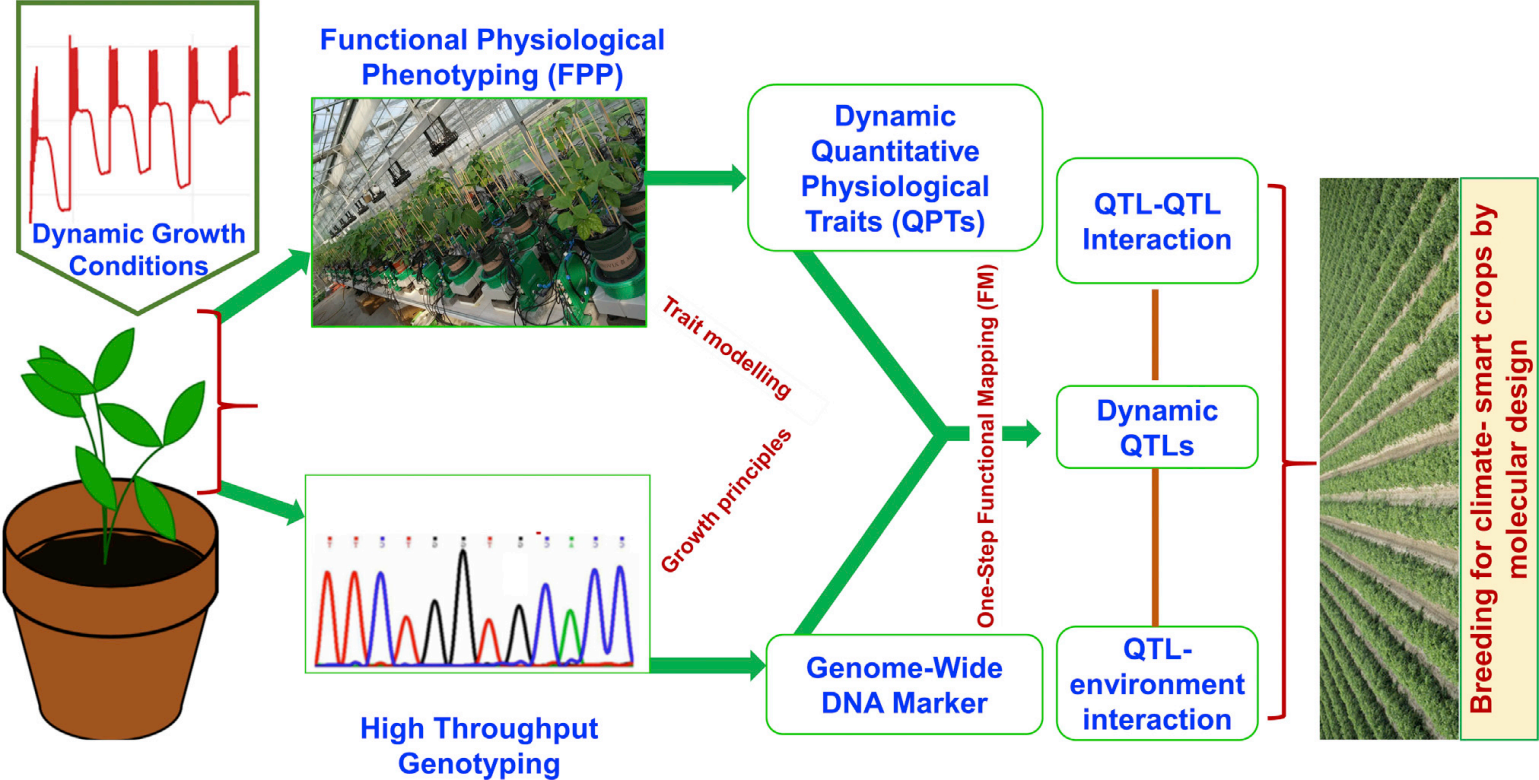
Schematic display of the principle of joint FPP-FM analysis As high-throughput genotyping has no longer been a bottleneck in the post-genome era, the cornerstones of the joint FPP-FM framework should include: (1) a high-throughput FPP platform enabling acquisition of dynamic quantitative physiological traits (QPTs) data from a natural or pedigree population. Exemplified here is the PlantArray system that collects dynamic data of environmental parameters (e.g. temperature, radiation), stress strength (soil water content) and system weight from hundreds of plants simultaneously, which are translated into critical whole-plant water-relation parameters (e.g. E, Gs, WUE) by using proved, physiology-based algorithms. (2) a powerful genetic framework in which dynamic QPTs data, together with genome-wide DNA marker information, are implemented into a one-step FM algorithm to reveal the QTLs behind the QPT plasticity and assess the interplay between QTLs or QTL-environment interactions over time. Through joint FPP-FM analysis, the dynamic effects of the QTLs, their interaction with specific environmental scenarios and the QTL network information that highlights QTL topology, organization, evolution and hub QTLs in the course of treatment are elucidated.

Here, we developed a novel framework called FPP-FM in which phenotypic data from functional physiological phenotyping (FPP) under multiple treatments, together with genotypic (marker) information, are implemented into a functional mapping (FM) algorithm in order to reveal the QTLs behind the QPT and assess the interplay between QTLs or QTL-environment interactions over time (Figure 1). FPP is a physiology-based, high-throughput, nondestructive, and non-invasive phenotyping technique that continuously measures the plant and its ambient conditions including soil and atmosphere (Gosa et al., 2019). By showcasing the application of FPP-FM to an introgression line population of tomato under progressive drought stress followed by recovery, we demonstrate the efficiency and power of this method in dissecting the genetic architecture of the dynamic trait of weight-normalized transpiration rate (E). Because the ideas of FPP and FM both are universal, this joint framework can be applied to other longitudinal traits related to development and responses to broader types of abiotic and biotic stresses.

## Results

### Functional physiological trait analysis

**Figure 2:**
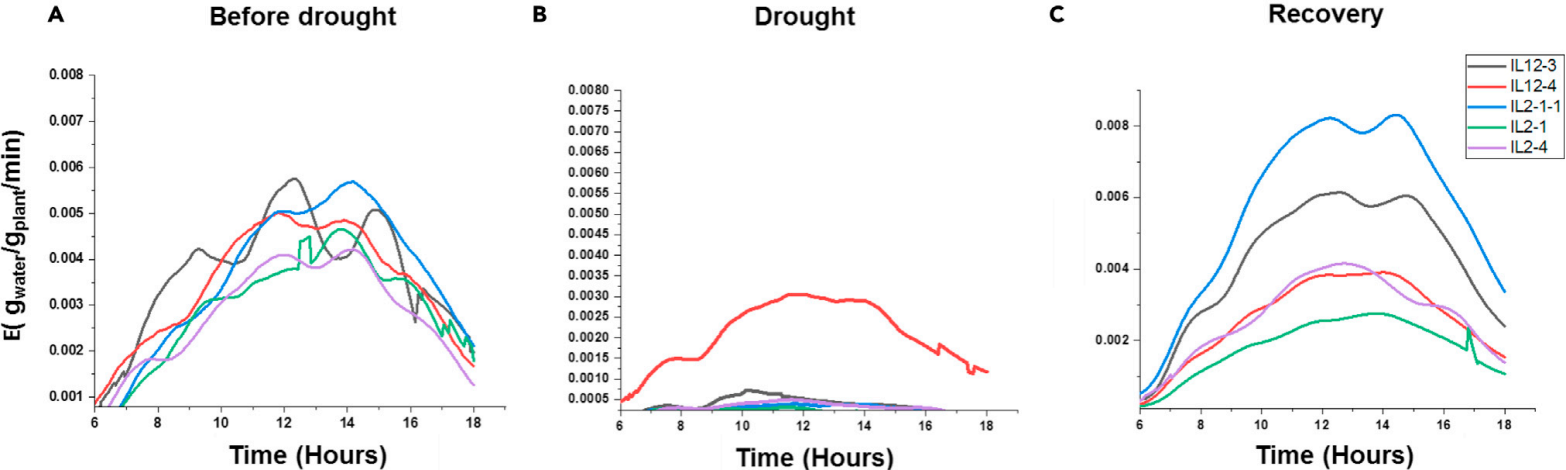
Graphic display of transpiration rate for five representatives ILs Data from the before-drought (A), drought-stress (B), and recovery (C) phases are shwon. Measurements were taken using the FPP platform PlantArray 3.0 system (Plant-DiTech, Rehovot, Israel). Plants were grown in 3.9-L pots filled with sand, in a partially controlled greenhouse in which the temperature was moderated by fans cooling a wet mattress and the plants were exposed to natural day length and light. The weight output of the load cells was monitored every 3 min. .

The first step of FPP-FM is to acquire dynamic quantitative physiological traits data from a natural or pedigreed population. Here, the population used was an introgression line population of tomato, which were subjected to a treatment scenario comprising normal irrigation, progressive drought, and water recovery in a high-throughput, automatic phenotyping system (Figure 1). During the entire course of this experiment, the phenotypic data of multiple drought-responsive traits such as plant weight and transcription rate were continually recorded alongside with the stress parameters. A detailed description of the FPP process for this population was already published by Halperin et al. (2017). For a reasonable computational intensity, here we used transpiration rate (Tr) as an example to show how our proposed framework translated the dynamic phenotypic information into genetic knowledge. Detailed phenotypic and genotypic data used in this study is deposited in Table S1 (source data). In Figures 2A–2C, the dynamic patterns of transpiration rate from five randomly selected IL lines were displayed, which were recorded every three minutes spanning the before-drought (Figure 2A), drought-stressed (Figure 2B), and the recovery phases (Figure 2C). Transpiration rate is known to associate with yield penalty under drought stress (De Wit, 1958; Sade et al., 2010). Upon water withholding, a profound reduction of transpiration rate was observed for all five lines (Figure 2B), whereas the genotypic differences in agility and magnitude of the response were apparent. Following water resumption, significant genotypic differences in the pattern of recovery were observed, which, however, were not correlated to the plant behaviors in response to water deficit. The line IL2-1-1, for example, maintained the highest transpiration rate under drought stress (Figure 2B), but its capacity to recover was weak following rewatering (Figure 2C).

### Fitting of multiphased phenotypic data and QTL mapping by functional mapping

**Figure 3:**
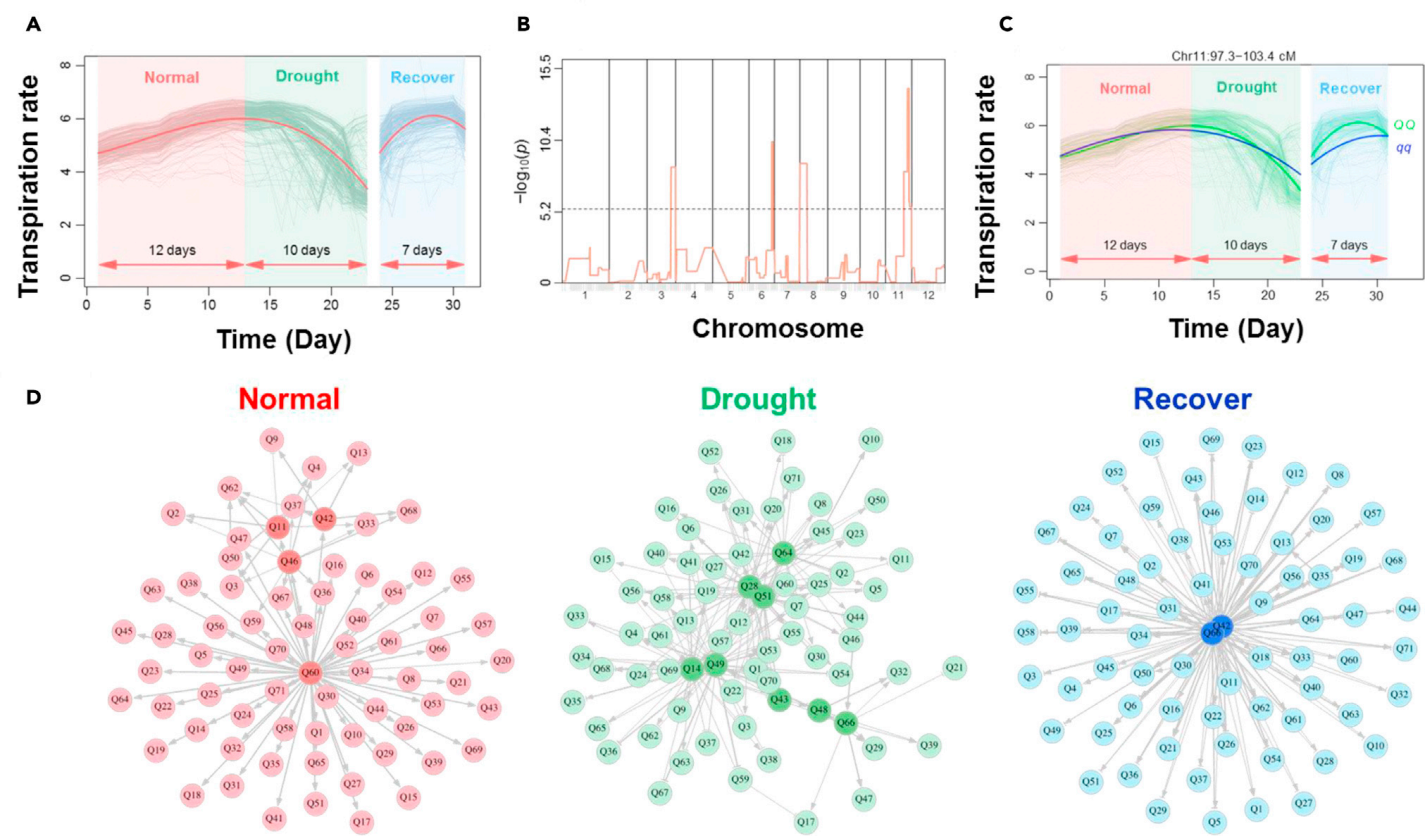
Dynamic QTLs and QTL networks controlling transpiration rate (A), longitudinal transpiration rates of all ILs whose mean curve (red) was fitted by Legendre function; (B), Manhattan plot of significances for the association of markers with dynamic changes of E across the tomato genome by FPP-FM. The dashed horizontal line indicates the critical threshold of p value at the 5% significance level determined by the permutation test. (C), dynamic effects of the two genotypes (*QQ* and *qq*) at the most significant QTL (chromosome 11) on transpiration rate. (D), QTL interaction networks in the pre-treatment, drought-stressed, and recovery phases. Each node denotes a QTL and each edge represents the interaction between two QTLs. The hub QTLs were highlighted with darkened colors in each network.

We implemented the Legendre function and the structured antedependence model (Zhao et al., 2005) to the phenotypic data. We modeled the QTL genotypic mean vector of transpiration rate and approximated the structure of the covariance matrix, which was a key step to integrate the fitted parameters into the functional mapping framework (see “STAR methods” for details). The mean curves of the normalized transpiration rate for all the ILs in nonstressed (day 0 to day 12), drought-stressed (day 13 to day 23), and recovery (day 24 to day 30) phases were demonstrated in Figure 3A. It clearly showed that, as a whole, in the first phase, the transpiration rate increased gradually as the plants grew; in the second phase, its trend turned to a gradual decrease; and in the third phase, the transpiration rate increased rapidly, which reached the maximum level on the fifth day after rewatering and was followed by a decrease since then.

Following incorporating the fitted parameters into functional mapping, we identified, in a single step, 7 large-effect QTLs under the threshold of p < 3.979 × 10^−6^ calculated by permutation test (Figure 3B). The most significant QTL was located on chromosome 11 (97.30–103.4 cM, *Qtr.11* hereafter). The effects of the two alleles (QQ and qq) at this QTL were found to show a major difference only in the third phase, suggesting that *Qtr.11* is a conditional QTL depending on the specific treatment scenario of rehydration (Figure 3C). Indeed, by further testing hypothesis 11 (see “STAR methods”), we revealed that the expression of *Qtr.11* in response to water recovery was through QTL-environment interaction to affect the transpiration rate (p < 0.01).

### Integration of QTLs to develop a network

We further reconstructed networks of QTLs that interact with each other to show how they confer adaptation of plants to environmental changes. The limited number (1,077) of discrete SNPs in the IL population due to the cosegregation of SNPs (2,521 in total) in each chromosome bins allowed us to reconstruct reasonably sized, full-scale networks to reveal the interactions among all QTLs with major or minor effects. Through modeling analysis, networks for the three treatment scenarios were constructed that in total included 71 nodes, with each node denoting a QTL and each edge representing the interaction between two QTLs. The shifts of nodes in different networks led to changes of the network topology in various scenarios, reflecting dynamic responses of QTL interactions to the environment. We identified four hub nodes, Q11, Q42, Q46, and Q60, under the normal (well-watered) condition, which modulated the genetic network of transpiration rate predominately by activating other QTLs. Under drought condition, 8 hub QTLs (Q64, Q28, Q51, Q14, Q49, Q43, Q48, and Q66) were detected, which played a role either by activating or repressing the expressions of other QTLs and were under positive or negative regulations by other core or peripheral QTLs themselves. We detected two hub nodes (Q66 and Q42) that interacted with other nodes to maintain the stability and dynamics of the recovery network. Highly interestingly, Q66 and Q42 were previously found in the drought and normal networks, respectively, and thus were considered to be plasticity QTLs linking normal transpiration and stress responses for balanced plant growth and drought tolerance upon water recovery. Because episodes of severe stress are often followed by periods of alleviated tension in field conditions, our findings of environment-depend QTLs, in particular, the discovery of the hub plasticity QTLs in the recovery phase, have great agronomic implications. Leveraging this core genetic knowledge gained from FPP-FM analysis would facilitate the modulation of plant fitness and resiliency through molecular breeding approaches, to ultimately achieve the goal of minimum yield loss under various drought conditions.

### Comparison with existing methods and simulation analysis

To our knowledge, there is no currently available tool like ours that is capable of fitting multiphased physiological data and being integrated with functional mapping. We conducted a Bayesian group lasso analysis and ANOVA with the same genotypic and phenotypic data to determine the extent to which the QTLs mapping was affected by the choice of methods. As a result, the Bayesian method detected only one QTL with a relative high p value (-log_10_*P* = 1.5, Figure S1) whereas the ANOVA was able to detect only two QTLs, one for the fourth day (-log_10_*P* = 10.2, Figure S2, Table S2) and the other for the 22^nd^ day (-log_10_*P* = 6.3, Figure S3, Table S3) of the experiment. The positions of the significant markers detected by different methods were consistent. These results demonstrate that FPP-FM was precise and more powerful in detecting QTLs from dynamic physiological data.

To further provide a theoretical estimate of the powers of FPP-FM and the traditional mapping method (ANOVA, for IL population), a simulation analysis was performed. As expected, ANOVA well detected significant QTLs from the uncorrelated data at a large sample size and heritability; however, its power for QTL detection in time-correlated data was substantially low (0.34–0.61; Table 1). In contrast, our new model showed higher power to detect QTLs underlying the time-correlated data, whereas its performance in handling time-uncorrelated data was similar to that of ANOVA (Table 1). The new model also showed much lower false-positive rate in QTLs detection in the simulation analysis, especially for the time-correlated data (1/2–1/7 of that from ANOVA, Table 2). To conclude, FPP-FM was proved to be a powerful new tool to identify specific QTLs that govern processes and patterns of physiological traits over time.

**Table 1:** Power of QTL detection by ANOVA and FPP-FM from the data simulated under different heritabilities (0.05 and 0.10), for the sample sizes 500 and 200

		Simulation							
		Time-uncorrelated time-correlated							
		N = 200		N = 500		N = 200		N = 500	
Method		H^2^ =	H^2^ =	H^2^ =	H^2^ =	H^2^ =	H^2^ =	H^2^ =	H^2^ =
		0.05	0.1	0.05	0.1	0.05	0.1	0.05	0.1
	**ANOVA**	74%	84%	86%	92%	34%	57%	42%	61%
	**FPP-FM**	72%	83%	88%	93%	78%	92%	91%	95%

**Table 2:** False positive rate of QTL detection by ANOVA and FPP-FM from the data simulated under different heritabilities (0.05 and 0.10), for the sample sizes 500 and 200

		Simulation							
		Time-uncorrelated time-correlated							
Method		N = 200		N = 500		N = 200		N = 500	
		H^2^ =	H^2^ =	H^2^ =	H^2^ =	H^2^ =	H^2^ =	H^2^ =	H^2^ =
		0.05	0.1	0.05	0.1	0.05	0.1	0.05	0.1
	**ANOVA**	22%	14%	11%	8%	45%	34%	31%	28%
	**FPP-FM**	19%	15%	9%	6%	23%	17%	11%	4%

## Discussion

The phenomics era is witnessing a burst of multiphased phenotypic data derived from various treatments. In order to leverage this advantage in breeding programs, the genetic architecture behind the traits must be elucidated to enable breeders to manipulate these traits with DNA markers for a large number of genotypes. To date, the contribution of the physiological trait-based selection of optimal drought response is minor (Ghanem et al., 2015) as compared with morphological traits (Sadras and Richards, 2014) largely because of the big challenges in genetic dissection of these traits (Easlon et al., 2014; Feldman et al., 2018). This has become a rate-limiting step in reaping the fruit of phenomics. For example, to increase carbon assimilation toward a higher yield, one may consider manipulating Gs or the biosynthesis of its regulator, ABA (Sinclair et al., 1984; Blum, 2009). However, as stomata is the gateway of both H_2_O and CO_2_, selection for stomata behavior needs to be very conditional and dynamic to balance water evaporation and carbon intake according to specific ambient conditions that typically related to geographic locations, cultivation seasons, and plant growth stages. Clearly, the success of harnessing quantitative physiological trait plasticity to improve agronomic drought resistance relies heavily on prior knowledge on QTLs governing the individual traits, and hence, it is imperative to map and identify a comprehensive set of QTLs that are involved in the ecophysiological dynamics of drought response during the plant's life cycle.

In this article, we propose that the principle of functional physiological phenotyping and that of functional mapping can be unified to provide a powerful tool to draw real-time pictures that illustrate the causal networks of genetic interactions along the short-term axis of physiological response to the environment and the long-term axis of development. By taking transpiration rate as an example, we demonstrated how the proposed FPP-FM framework help to better unravel and comprehend the genetic architecture of phenotypic formation and plasticity. As bridging the phenotype-genotype gap is at the core of advancing our mechanistic understanding of phenotypic complexities, alterations, and novelties, our conceptual framework potentially provides an engine for this aim and specifically offers a novel physiological-genetic-environmental functional strategy to dissect the main contributors to yield stableness. We implement the idea of FM (Wu and Lin, 2006) to map multitreatment physiological data in time-series by combining multistage nonparametric equation and high-dimensional covariance matrix. Our approach detects and estimates genetic effects due to allelic/nonallelic actions and QTL interactions from physiological and developmental principles of growth. The simulation study clearly showed an over performance of our approach to the traditional static mapping for longitudinal phenotypic data. By choosing an optimal order of LOP via information criteria, our approach overcomes the overfitting or underfitting of multiphased physiological data. The approach can reconstruct QTL networks, providing a powerful tool to understand how QTL-QTL and QTL-environment interactions affect trait formation in a set of specific environmental scenarios and thereby allows for identifying the hub QTLs in network topology, organization, and function.

In the demonstrative case study here, a tomato pedigreed population was analyzed; if a mapping population is constructed using a natural population, the theory of linkage disequilibrium mapping should be integrated within the functional mapping framework to provide a powerful tool for fine-scale mapping of complex traits (Lou et al., 2003). Therefore, the extension of our model to tackle genotype-environment interactions using genome-wide association study is straightforward. It is also noteworthy that the limited capacity of physiological phenotyping and the challenge in acquiring difficult-to-measure traits like photosynthesis still exists, which restricts the immediate use of our method to larger and richer types of populations. However, the highly uniform environments (hence less noises) in this kind of experiments would reduce the size requirement of population in genetic mapping. More importantly, with the rapid deployment of phenotyping facilities all over the world along with their growing phenotyping capacity (e.g. more number of units, smaller size) and the successful integration of lysimeters and imaging system (Weksler et al., 2020), FPP-FM will show its usefulness in serving the translation of physiological data into genetic gain. In principle, our method is extendable to morphological traits showing plasticity and dependence on treatment because the concept of functional mapping is general and the idea of integrating dynamic phenotypes into functional mapping is also general. Equation (1) is a redundant joint likelihood, thus can be extended to consider multiple phenotypes at once. Nevertheless, because different types of traits are usually captured by different phenotyping systems, a case-by-case modification of the fitting algorithm for the phenotype will be needed.

The proposed FPP-FM framework can further be integrated with molecular biology approaches such as transcriptomic, proteomic and epigenetic profiling as well as gene/protein coexpression networking, to more deeply and systematically reveal how the plant water status and yield are affected by the interplay between genetic, epigenetic, and environmental factors. In such an effort, greater biological relevance will be achieved if the molecular analysis is guided by the principle of FPP because it allows for sampling the plant tissues based on physiological status rather than time of treatment. It is therefore expected to offer unprecedented opportunities to reveal the molecular mechanisms underlying genotypic differences of plant responses to the environment. From the perspective of breeding, FPP-FM is also a useful tool. First, it provides markers linked to yield-related physiological traits, which will allow for earlier stage, more direct, and efficient selection of the genes regulating transpiration and photosynthesis under drought stresses. Second, the dissection of the dynamic expression of the QTLs, i.e. when they are expressed as the stress scenario evolves, is valuable for the molecular design of crops adaptive to a certain ecoclimatic condition. For example, in the current case, the QTL *Q66* may represent a good selection target in areas featured by intermittent drought, provided that this QTL plays a role in balancing drought response and plant growth; however, in long-term, severe drought-prone areas, QTLs that confer stably conservative water use may be preferred.

### Limitations of the study

As aforementioned, the limited capacity of physiological phenotyping in many current platforms and the still-existing challenge in acquiring difficult-to-measure traits like photosynthesis are the major factors restricting the immediate use of our method to larger populations. Also, despite the principle of our method is general, the application of it to traits that are captured by other phenotyping systems may need a case-by-case modification of the fitting algorithm.

## STAR★Methods

### Key resources table

**Table:** 

REAGENT OR RESOURCE	SOURCE	IDENTIFIER
R software		https://github.com/FFP-FM/Version1

### Resource availability

#### Lead contact

Further information and requests for resources and reagents should be directed to and will be fulfilled by the Lead Contact: Prof. Pei Xu (peixu@cjlu.edu.cn)

#### Materials availability

This study did not generate new unique reagents.

#### Data and code availability

All statistical algorithms that build our framework into a user-friendly R package for public use are available on https://github.com/FFP-FM/Version1.

### Experimental model and subject details section

An introgression line (IL) population of tomato composed of 62 lines were used to determine whole-plant transpiration rates using an array of lysimeters placed in the greenhouse (Plantarray 3.0 system; Plant-DiTech) in the “iCORE Center for Functional Phenotyping” (http://departments.agri.huji.ac.il/plantscience/icore.phpon). We followed the Bayesian group lasso method with associated MCMC algorithms as developed by Li et al. (2015). ANOVA is a common (and “static”) method used to detect associations between molecular markers and traits in IL populations.

### Method details

#### Plant materials and growth conditions

In 2018, an introgression line (IL) population of tomato composed of 62 lines (Eshed and Zamir, 1995) were used to determine whole-plant transpiration rates (Table S1) using an array of lysimeters placed in the greenhouse (PlantArray 3.0 system; Plant-DiTech) in the “iCORE Center for Functional Phenotyping” (http://departments.agri.huji.ac.il/plantscience/icore.phpon), as described in details by Halperin et al. (2017). PlantArray combines gravimetric system, soil and atmospheric probes, controller and irrigation valves in a unit, which can measure or calculate plant water relation traits as well as the soil-atmosphere parameters at the same time, during the whole plant growth process. Briefly, plants were grown in 3.9L-pots filled with an inert substance (quartz sand) under semi-controlled temperature conditions (20-34°C day and 18-24°C night temperature) with natural light. Pots were placed on a temperature-compensated load cell with digital output (**Vishay Tedea Hunt Leigh**). Plants were grown in a circle cut out of white plates that is used to prevent evaporation. We irrigated pots at night in multiple irrigations (3 minutes irrigation, every 15 minutes for 6 hours) during the pre-drought treatment period with dripper assemblies **(Netafim, Israel**) pushed into the sand to ensure the medium was evenly wet. Once the plants reach high daily transpiration (above 400g/day), drought treatment was started by gradually irrigating plants at 60% of their daily transpiration while allowing all plants to gradually experience water stress. After each plant reached its θ_crit_, the point at which the availability of soil water becomes a limiting factor to transpiration rate, the irrigation was resumed to evaluate the recovery of each genotype. The SNPs genotypic information (Table S1) of this population was retrieved from Sim et al. (2012) and used during analysis.

#### Whole-plant transpiration and soil-atmospheric measurements

The PlantArray 3.0 platform is a multi-sensor telemetric platform that enables simultaneous and continuous monitoring of key plants' physiological traits. A detailed description of the system was recently published in Dalal et al. (2020). Briefly, the system consists of highly sensitive, temperature compensated load cells that are used as a weighing lysimeter. A lysimeter is a measuring device that can be used to measure the amount of actual transpiration which is released by plants from a monotonic weight reduction throughout the day. The amount of water loss by transpiration can be worked out by calculating the difference between the weight before and after irrigation. The water loss is the phase of daily transpiration which was calculated from the weight difference between the two data points for individual plants. The plant weight was calculated as the difference between the total system weight and the sum of the tare weight of pot plus drainage container, the weight of soil at pot capacity, and weight of water in the drainage container at the end of the free drainage (Halperin et al., 2017; Gosa et al., 2019). By using soil weight and initial plant weight, a set of phenotypic data can be extracted including data on growth rates, transpiration, canopy conductance, and soil water content. Soil-atmosphere parameters including temperatures, relative humidity, radiation, and soil moisture were monitored by using the LI-COR 190 Quantum Sensor (**Lincoln, NE, USA**) and the soil moisture sensor (**5TE; Decagon Devices, Pullman, WA, USA**), respectively. The measurement was made every 3 minutes. The data of soil-atmosphere parameters and plant growth and physiological parameters including plant weight, daily transpiration, transpiration rate (Tr) and normalized transpiration (E) were simultaneously saved on the online web-based software SPAC analytics (**Plant-Ditech, Israel**). The data of whole plant transpiration rate (Tr) was used in the following genetic analysis.

#### Statistical model for functional mapping

##### Joint likelihood

Let y_*il*_=(y_*il*_($t_{1il}^{1}$), ...., y_*il*_($t_{Til}^{1}$), y_*i*_($t_{1il}^{2}$), ...., y_*i*_($t_{Til}^{2}$), ..., y_*i*_($t_{1il}^{s}$), ...., y_*i*_($t_{Til}^{s}$)) denotes the phenotypic value of a trait *l* (*l*=1*, ..., L*) for individual *i* at different time points under treatment *s* (*s=*1*, ..., S*) from a natural or controlled population. Note that ($t_{1il}^{s}$, ..., $t_{Til}^{s}$) represents a series *T* time points which reflects the treatment stage such as drought or recovery. At a given QTL with the *j*th genotype having *n*_*j*_ individuals, the joint likelihood of phenotypic values at this marker is expressed as

(Equation 1) $$L=\prod_{l=1}^{L}\prod_{j=1}^{J}\prod_{i=1}^{n_{j}}f_{lj}\left(y_{li};\mu_{lj},\Sigma_{i}\right)$$

where flj(yli;μlj,Σ)i is a multivariate normal distribution with genotype-specific mean vector over different traits, expressed as

(Equation 2) $$\mu_{lj}=\left(\mu_{lj}^{1},\mu_{lj}^{2},..,\mu_{lj}^{S}\right)=\left(\mu_{lj}\left(t_{1l}^{1}\right),...,\mu_{lj}\left(t_{Tl}^{1}\right),\mu_{lj}\left(t_{1l}^{2}\right),...,\mu_{lj}\left(t_{Tl}^{2}\right),...,\mu_{lj}\left(t_{1l}^{S}\right),...,\mu_{lj}\left(t_{Tl}^{S}\right)\right)$$

and covariance matrix is expressed as

(Equation 3) $$\Sigma =\left[\begin{array}{lll} \Sigma_{1} & \cdots & \Sigma_{1l}\\ \vdots & \ddots & \vdots \\ \Sigma_{l1} & \cdots & \Sigma_{l} \end{array}\right]$$

where $\Sigma_{l}$ is the residual covariance matrix for trait *l*, $\Sigma_{1l}=\Sigma_{l1}$ is the longitudinal covariance matrix between the two traits.

##### Modeling the genotype-specific mean vector

Functional mapping models the genotypic means for an assumed QTL at different time points by Legendre Orthogonal Polynomials (LOP). This function can fit any kind of curve (Lin and Wu, 2006). The general form of the Legendre polynomial of order *k* implemented to model the mean value of *j*th genotype for a marker is generally expressed as

(Equation 4) $$P_{lsk}\left(t_{Tl}^{s}\right)\;=\left(P_{ls0}\left(\tau_{Tl}^{s}\right),P_{ls1}\left(\tau_{Tl}^{s}\right),...,P_{lsk}\left(\tau_{Tl}^{s}\right)\right)\;$$

where

(Equation 5) $$P_{lsk}\left(\tau_{Tl}^{s}\right)=\frac{1}{2^{k}}\sum_{r=0}^{k/2}\frac{{\left(-1\right)}^{r}\left(2k-2r\right)!}{r\text{!}\left(k-r\right)\text{!}\left(k-2r\right)!}{\left(\tau_{Tl}^{s}\right)}^{k-2r}$$

where *k* = 0, 1, ..., *K* and $\tau_{Tl}^{s}=-1+\frac{2\left(t_{Tl}^{s}-\min\left(t_{Tl}^{s}\right)\right)}{\max\left(t_{Tl}^{s}\right)-\min\left(t_{Tl}^{s}\right)}$ with min($t_{Tl}^{s}$) and max($t_{Tl}^{s}$) being, respectively, the first and last time for the trait *l* under stage *s*. We rewrite the genotype-specific mean vectors as

(Equation 6) $$\mu_{lj}=\left(\mu_{lj}^{1},\mu_{lj}^{2},..,\mu_{lj}^{S}\right)=\left(P_{l1k}\left(t_{Tl}^{1}\right)v_{l1j}^{\prime }\text{,}P_{l2k}\left(t_{Tl}^{2}\right)v_{l2j}^{\prime }\text{,...,}\;P_{lsk}\left(t_{Tl}^{s}\right)v_{lsj}^{\prime }\right)$$

where $v_{lsj}=\left(v_{ls0j},v_{ls1j},...v_{lskj}\right)$ is a vector of genotypic values. By maximizing likelihood (1), time-invariant genotypic values $v_{lsj}$ for the longitudinal trait are estimated.

##### Modeling the covariance matrix

The covariance matrix is modeled by structured antedependence (SAD). SAD models autocorrelate structure of the covariance without relying on a stationary assumption of time-dependent variance and correlation (Zhao et al., 2005). The first-order SAD (SAD(1)) only considers the dependence of errors at the immediate time point, which can be generally applied to model the longitudinal changes of variances and covariances. The residual variance at time *t* and residual covariance between two times *t*_1_ and *t*_2_ can be derived as

(Equation 7) $$\begin{array}{l} \sigma^{2}\left(t\right)=\left[\frac{1-\rho^{2t}}{1-\rho^{2}}\right]\varphi^{2}\\ \sigma \left(t_{1},t_{2}\right)=\rho^{t_{2}-t_{1}}\left[\frac{1-\rho^{2t_{1}}}{1-\rho^{2}}\right]\varphi^{2} \end{array}$$

where $\varphi^{2}$ as the innovation variance at a different time is the constant, and ρ is the degree of antedependence.

##### Model selection

To obtain the best fit of the data, the optimal order of the Legendre polynomial to model the genotypic mean vector should be determined. One can use the AIC information criterion as the model selection criterion for the polynomial order. The AIC is defined as

(Equation 8) $$\text{AIC}=-2\;\ln\;L\left(\hat{\mu },\hat{\Sigma }\left|k\right)+2\dim\text{ension }\left(\hat{\mu },\hat{\Sigma }\right|k\right)$$

where $\left(\hat{\mu },\hat{\Sigma }|k\right)$ is the MLE of parameters for the Legendre polynomial of order *k* and dimension $\left(\hat{\mu },\hat{\Sigma }|k\right)$ represents the number of independent parameters under order *k*. The optimal model displays the minimum AIC value.

##### Computing algorithm

It is difficult to derive a closed-form of genotype-specific and covariance structure parameters; due to this reason, Nelder-Mead Simplex or the Quasi-Newton algorithm is used to estimate curve equation as well as the variance-covariance structuring parameters. These two methods do not depend on explicit equations that are implemented.

#### Hypothesis tests

##### Existence of a QTL

Under likelihood (1), whether there exists a QTL for the longitudinal phenotypes can be tested by formulating the null and alternative hypotheses as follows

(Equation 9) $$\begin{array}{l} H_{\text{0}}\text{: }v_{lsj}\equiv v_{ls}\text{ for }l=1\text{,..., }L\text{; }s=1\text{,..., }S\text{; }j=1\text{,..., }J\\ H_{1}\text{: Not all equalities in the }H_{\text{0}}\text{ hold} \end{array}$$

under each of which, the likelihoods are used to calculate a log-likelihood ratio which is compared against a critical threshold. If the null hypothesis above is rejected, a significant QTL is claimed. By reshuffling the phenotypic data for 1000 times, the critical threshold can be determined from permutation tests. At each time, an LR value was calculated and the top 5% of LR values among 1000 times was then chosen as the critical threshold at the 5% significance level.

##### Pleiotropic effect of a QTL

If a significant QTL exists, then we can test whether this QTL has a pleiotropic effect for different traits. This test is formulated as

(Equation 10) $$\begin{array}{l} H_{\text{0}}\text{: }v_{lsj}\equiv v_{sj}\text{ for }l=1\text{,..., }L\text{; }s=1\text{,..., }S\text{; }j=1\text{,..., }J\\ H_{1}\text{: Not all equalities in the }H_{\text{0}}\text{ hold} \end{array}$$

If the null hypothesis is rejected, this implies that the significant QTL detected affects pleiotropically longitudinal traits.

##### QTL by environment interaction

Further, we can test whether the QTL effect on the longitudinal trait is due to QTL × Environment interaction. This can be tested for different cues using

(Equation 11) $$\begin{array}{l} H_{\text{0}}\text{: }v_{lsj}\equiv v_{lj}\text{ for }l=1\text{,..., }L\text{; }s=1\text{,..., }S\text{; }j=1\text{,..., }J\\ H_{1}\text{: Not all equalities in the }H_{\text{0}}\text{ hold} \end{array}$$

The rejection of the above null hypothesis means that a significant genotype by environment interaction exists due to allelic sensitivity to a varying environment.

The LR for these hypothesis tests (Equations 10 and 11) are thought of as being chi-square distributed with the degrees of freedom equal to the difference in the number of parameters to be estimated under the alternative and null hypotheses. *P*-value was calculated by the chi-square distribution function for each LR, in which less than 0.05 was considered statistically significant. When these tests (Equations 10 and 11) were undertaken, bonferroni correction was used to control false positives.

##### QTL network reconstruction

For a given QTL of quantitative trait, its time-varying genetic variance is expressed as:

(Equation 12) $${V\left(t\right)=2p_{1}p_{0}\left(a+\left(p_{1}-p_{0}\right)d\right)}^{2}+4p_{1}^{2}p_{0}^{2}d^{2}$$

where *p*_1_ and *p*_0_ are allele frequency for two alleles, respectively; a and d are the estimate of the additive and dominant effect for QTL, respectively. Here, we implement a procedure to reconstruct a genetic network among all significant QTLs based on their genetic variance. Let *s*_*j*_(*t*)=$\sqrt{V_{j}\left(t\right)}$denote the genetic standard deviation of SNP *j* (*j*=*1*, ..., *J*) at time *t*. We construct a *J*-dimensional ordinary differential equations:

(Equation 13) $$\frac{ds_{j}\left(t\right)}{dt}=W_{j}\left(g_{j}\left(t\right):\Theta_{j}\right)+\sum_{j^{\prime }=1,j^{\prime }\neq j}^{J}W_{jj^{\prime }}\left(g_{j^{\prime }}\left(t\right):\Theta_{jj^{\prime }}\right)$$

where $W_{j}\left(g_{j}\left(t\right):\Theta_{j}\right)$ describes the main effect of this QTL that is expressed independently from the effects of any other QTLs, and $\sum_{j^{\prime }=1,j^{\prime }\neq j}^{J}W_{jj^{\prime }}\left(g_{j^{\prime }}\left(t\right):\Theta_{jj^{\prime }}\right)$ reflects the interactive effects of different QTLs on QTL j. $W_{j}\left(g_{j}\left(t\right):\Theta_{j}\right)$ and $\sum_{j^{\prime }=1,j^{\prime }\neq j}^{J}W_{jj^{\prime }}\left(g_{j^{\prime }}\left(t\right):\Theta_{jj^{\prime }}\right)$ are fitted by the nonparametric function such as B-spline or LOP. By estimate a set of ODE parameters $\Theta_{j}$, we can determine the pattern and magnitude of the independent effect of QTL. Meanwhile, the estimation of another set of ODE parameters $\Theta_{jj^{\prime }}$ enables us to characterize whether and how the effect of a QTL depends jointly on all other QTLs. Networks were visualized by using the package *igraph* in R software (Csardi and Nepusz, 2006).

### Quantification and statistical analysis

#### QTL analysis with existing methods

The performance of FPP-FM in detecting QTL from multiple-treatment longitudinal data was compared with those of the Bayesian method and one-way ANOVA. We followed the Bayesian group lasso method with associated MCMC algorithms as developed by Li et al. (2015). ANOVA is a common (and “static”) method used to detect associations between molecular markers and traits in IL populations. The same set of 2,521 SNPs markers (Sim et al., 2012) was used during analysis.

#### Simulation analysis

We conducted a computer simulation to validate the statistical properties of FPP-FM based on the working example used in this study. Data were simulated by assuming that traits are either correlated (using FPP-FM) or uncorrelated (using ANOVA, which is suitable for IL population) at different time points. The phenotype was determined by a set of QTLs among 1,000 simulated markers, plus a residual error following a multivariate normal distribution. We adjusted the innovation variance to obtain curve heritability levels of 0.05 and 0.10. The simulation considered two sample sizes: 500 and 200. Data were analyzed reciprocally using ANOVA and FPP-FM. For each simulated QTL, the estimate for the QTL parameter was the average of the corresponding estimates from the counted samples. The number of QTLs detected in every 1,000 simulated datasets represents the detection power of the QTL.

### Data and software package

The phenotypic and genotypic data of 62 introgression line (IL) population of tomato are available in Table S1. The genetic mapping data of E using Bayesian (Figure S1) and ANOVA (Tables S2 and S3, Figures S2 and S3) methods are available as supplementary files. We have coded all statistical algorithms that build our framework into a user-friendly R package for public use (https://github.com/FFP-FM/Version1).

## Supplemental information

Document S1. Figures S1–S3 and Tables S2 and S3

Table S1. Data of transpiration rate in 62 IL lines recorded by the PlantArray system, related to Tables 1 and 2 Genotypic and markers information of all ILs of tomato population
