# Supplementary material for: Functional physiological phenotyping with functional mapping: A general framework to bridge the phenotype-genotype gap in plant physiology
Source: iScience. 2021 Jul 10;24(8):102846. doi: 10.1016/j.isci.2021.102846 (PMC8333144; doi:10.1016/j.isci.2021.102846)
Supplement: Document S1. Figures S1–S3 and Tables S2 and S3 [file mmc1.pdf]

iScience, Volume *24*

Supplemental information

Functional physiological phenotyping with functional mapping: A general framework to bridge the phenotype-genotype gap in plant physiology

Arun K. Pandey, Libo Jiang, Menachem Moshelion, Sanbon Chaka Gosa, Ting Sun, Qin Lin, Rongling Wu, and Pei Xu

Supplemental Information

FPP-FM: a general framework to bridge the phenotype-genotype gap in plant physiology

Arun K Pandey[1], Libo Jiang[2], Menachem Moshelion[3,*], Sanbon Chaka Gosa[3], Ting Sun[1], Qin Lin[4], Rongling Wu[5,*], Pei Xu[1, 6*]

Supplemental figure

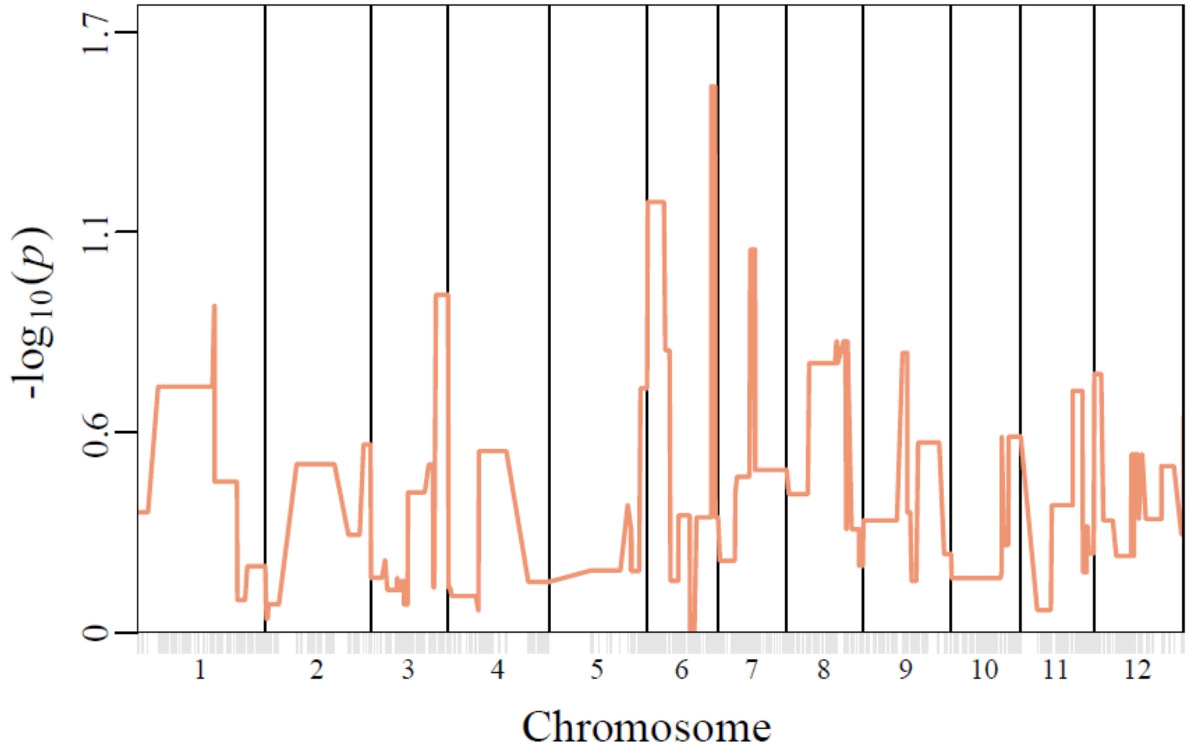

Figure S1: Result of Bayesian methods, Related to Figure 2. The Bayesian method detected only one QTL with a relative high *P*-value ($-\log_{10}P$=1.5)

Supplemental figure

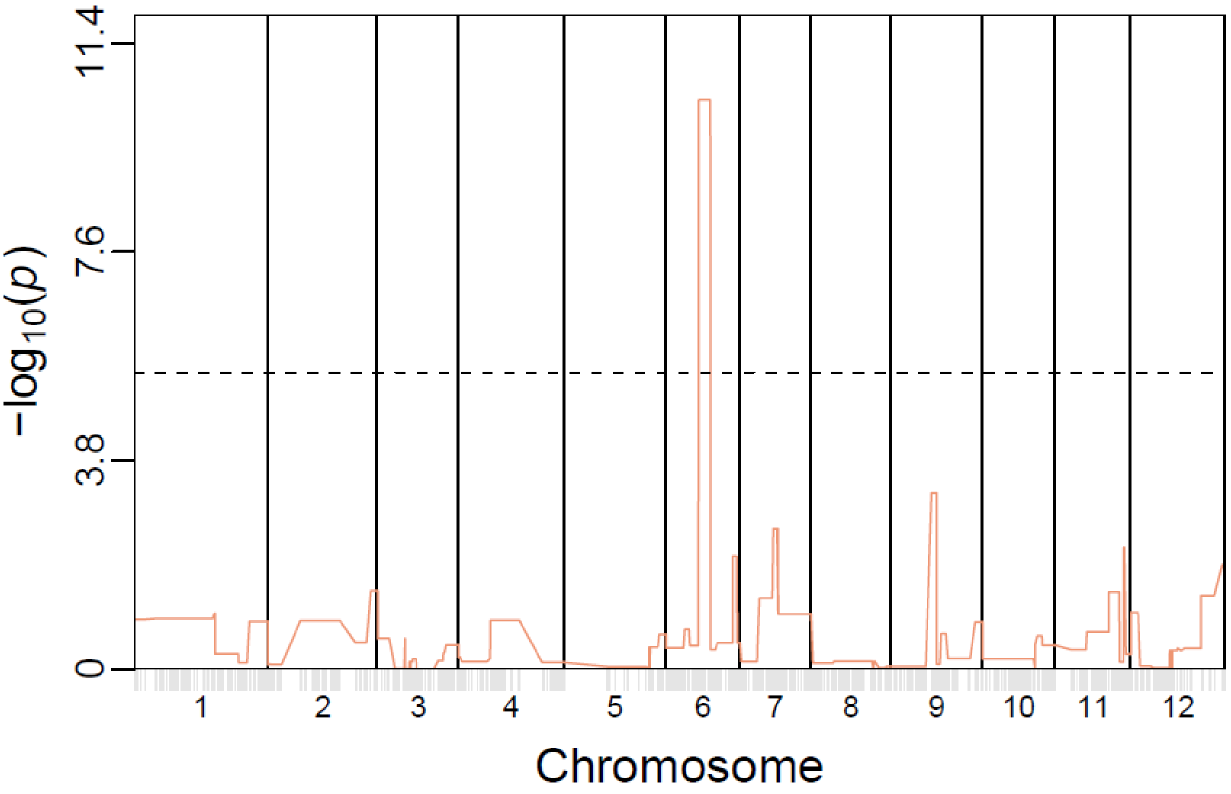

Figure S2: Result of ANOVA_4th day experiment, Related to Figure 2. The ANOVA was able to detect QTLs for the 4th day ($-\log_{10}P = 10.2$) of the experiment.

Supplemental figure

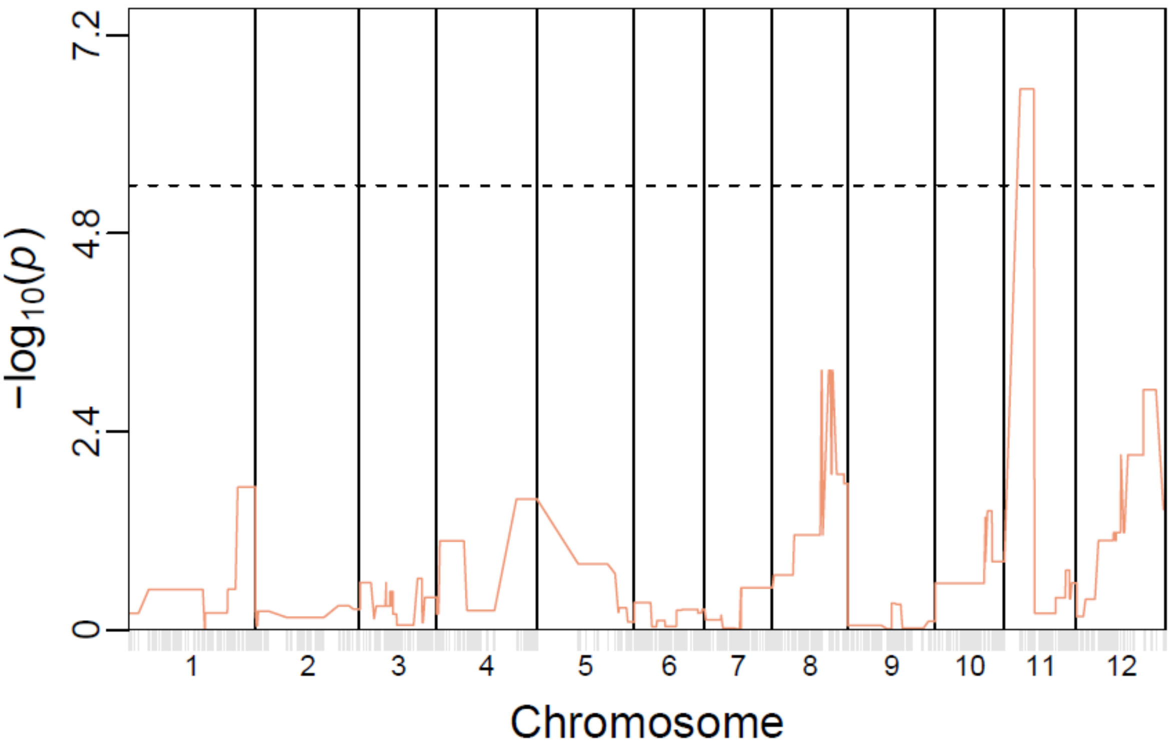

Figure S3: Result of ANOVA_22nd day experiment, Related to Figure 2. The ANOVA was able to detect only QTLs for 22nd day ($-\log_{10}P = 6.3$) of the experiment.

Supplemental table

Table S1. Data of transpiration rate in 62 IL lines recorded by the PlantArray system, Related to Tables 1 and 2. Genotypic and markers information of all ILs of tomato population (Submitted in Excel sheet)

Supplemental table

Table S2: Result of ANOVA_4th day experiment, Related to Tables 1 and 2. The Marker information on days 4 from ANOVA analysis

	SNP.Marker	Chromosome	Position (cM)	pvalue
1385	CL015656-0336	6	48.8	4.12E-11
1386	solcap_snp_sl_39064	6	49.5	4.12E-11
1387	solcap_snp_sl_39073	6	49.5	4.12E-11
1388	solcap_snp_sl_39159	6	51.5	4.12E-11
1389	solcap_snp_sl_39163	6	51.5	4.12E-11
1390	solcap_snp_sl_39246	6	52.2	4.12E-11
1391	solcap_snp_sl_39312	6	54.9	4.12E-11
1392	solcap_snp_sl_57866	6	56.9	4.12E-11
1393	solcap_snp_sl_57810	6	57.6	4.12E-11
1394	solcap_snp_sl_57766	6	58.3	4.12E-11
1395	solcap_snp_sl_57714	6	59.6	4.12E-11
1396	solcap_snp_sl_19915	6	63	4.12E-11
1397	solcap_snp_sl_24454	6	64.3	4.12E-11
1398	solcap_snp_sl_57607	6	64.3	4.12E-11
1399	solcap_snp_sl_24450	6	66.4	4.12E-11
1400	solcap_snp_sl_57594	6	66.4	4.12E-11
1401	solcap_snp_sl_57593	6	66.4	4.12E-11
1402	SL10882_924	6	66.4	4.12E-11
1403	solcap_snp_sl_24449	6	66.4	4.12E-11

Supplemental table

Table S3: Result of ANOVA_22nd day experiment, Related to Tables 1 and 2. The Marker information on days 22 from ANOVA.

	SNP.Marker	Chromosome	Position (cM)	pvalue
2096	solcap_snp_sl_66232	11	25	2.68E-07
2097	SGN-U581948_snp19879	11	25	2.68E-07
2098	SGN-U563067_snp11442_solcap_snp_sl_66221	11	25	2.68E-07
2099	solcap_snp_sl_66193	11	25.7	2.68E-07
2100	solcap_snp_sl_21767	11	29.8	2.68E-07
2101	solcap_snp_sl_66088	11	29.8	2.68E-07
2102	solcap_snp_sl_34253	11	29.8	2.68E-07
2103	solcap_snp_sl_34264	11	33.2	2.68E-07
2104	CL017088-0339	11	33.2	2.68E-07
2105	solcap_snp_sl_21059	11	33.8	2.68E-07
2106	solcap_snp_sl_21102	11	36.5	2.68E-07
2107	solcap_snp_sl_21109	11	36.5	2.68E-07
2108	solcap_snp_sl_21115	11	36.5	2.68E-07
2109	solcap_snp_sl_21116	11	36.5	2.68E-07
2110	solcap_snp_sl_21118	11	37.2	2.68E-07
2111	solcap_snp_sl_21119	11	37.2	2.68E-07
2112	solcap_snp_sl_21120	11	37.2	2.68E-07
2113	solcap_snp_sl_21127	11	37.2	2.68E-07
2114	CL017248-0137	11	37.2	2.68E-07
2115	solcap_snp_sl_9441	11	37.2	2.68E-07
2116	solcap_snp_sl_9443	11	37.2	2.68E-07
2117	solcap_snp_sl_9444	11	37.2	2.68E-07
2118	solcap_snp_sl_9445	11	37.2	2.68E-07
2119	solcap_snp_sl_9446	11	37.2	2.68E-07
2120	solcap_snp_sl_9447	11	37.2	2.68E-07
2121	solcap_snp_sl_9450	11	37.2	2.68E-07
2122	solcap_snp_sl_15247	11	37.2	2.68E-07
2123	solcap_snp_sl_15248	11	37.2	2.68E-07
2124	solcap_snp_sl_62695	11	37.2	2.68E-07
2125	solcap_snp_sl_9462	11	37.2	2.68E-07
2126	solcap_snp_sl_9470	11	37.2	2.68E-07
2127	solcap_snp_sl_9471	11	37.2	2.68E-07
2128	solcap_snp_sl_9481	11	37.2	2.68E-07
2129	CL015453-0346	11	37.9	2.68E-07

2130	solcap_snp_sl_62690	11	37.9	2.68E-07
2131	solcap_snp_sl_62702	11	39.2	2.68E-07
2132	solcap_snp_sl_9484	11	39.2	2.68E-07
2133	solcap_snp_sl_62748	11	41.9	2.68E-07
2134	solcap_snp_sl_62778	11	43.9	2.68E-07
2135	solcap_snp_sl_9507	11	43.9	2.68E-07
2136	solcap_snp_sl_9509	11	43.9	2.68E-07
2137	solcap_snp_sl_15269	11	43.9	2.68E-07
2138	solcap_snp_sl_9511	11	44.6	2.68E-07
2139	solcap_snp_sl_9516	11	44.6	2.68E-07
2140	solcap_snp_sl_9517	11	44.6	2.68E-07
2141	solcap_snp_sl_9519	11	44.6	2.68E-07
2142	solcap_snp_sl_62807	11	44.6	2.68E-07
2143	solcap_snp_sl_62808	11	44.6	2.68E-07
2144	solcap_snp_sl_9528	11	44.6	2.68E-07
2145	solcap_snp_sl_9529	11	44.6	2.68E-07
2146	solcap_snp_sl_9530	11	44.6	2.68E-07
2147	solcap_snp_sl_9533	11	44.6	2.68E-07
2148	solcap_snp_sl_9538	11	44.6	2.68E-07
2149	solcap_snp_sl_9545	11	44.6	2.68E-07
2150	solcap_snp_sl_9549	11	44.6	2.68E-07
2151	solcap_snp_sl_9550	11	44.6	2.68E-07
2152	solcap_snp_sl_9552	11	44.6	2.68E-07
2153	solcap_snp_sl_9556	11	44.6	2.68E-07
2154	solcap_snp_sl_9560	11	44.6	2.68E-07
2155	solcap_snp_sl_62822	11	44.6	2.68E-07
2156	solcap_snp_sl_62843	11	44.6	2.68E-07
2157	CL015007-0405	11	44.6	2.68E-07
2158	CL015225-0397	11	44.6	2.68E-07
2159	solcap_snp_sl_62864	11	45.3	2.68E-07
2160	CL017668-0415	11	46	2.68E-07
2161	solcap_snp_sl_34336	11	46.6	2.68E-07
2162	CL016994-0123	11	46.6	2.68E-07
2163	CL017534-0356	11	46.6	2.68E-07
2164	solcap_snp_sl_24976	11	46.6	2.68E-07
2165	solcap_snp_sl_24977	11	46.6	2.68E-07
2166	CL016728-0598	11	46.6	2.68E-07
2167	solcap_snp_sl_24979	11	46.6	2.68E-07
2168	solcap_snp_sl_34348	11	46.6	2.68E-07
2169	solcap_snp_sl_24981	11	46.6	2.68E-07
2170	solcap_snp_sl_24982	11	46.6	2.68E-07

2171	solcap_snp_sl_24983	11	46.6	2.68E-07
2172	solcap_snp_sl_24986	11	46.6	2.68E-07
2173	solcap_snp_sl_24987	11	46.6	2.68E-07
2174	solcap_snp_sl_24988	11	46.6	2.68E-07
2175	solcap_snp_sl_24985	11	46.6	2.68E-07
